# Use of the processes vaginalis: A new technique for reinforcing the neourethra in hypospadias associated with undescended testis

**DOI:** 10.4103/0970-1591.56187

**Published:** 2009

**Authors:** Himanshu Acharya, Shivaji B. Mane, Nitin P. Dhende, Abu Obeidah

**Affiliations:** Department of Paediatric Surgery, Sir J J Group of Hospitals, Mumbai, India

**Keywords:** Hypospadias, processes vaginalis, undescended testis, urethrocutaneous fistula

## Abstract

**Context:**

The incidence of undescended testis (UDT) along with hypospadias varies from 6 to 31%. The simultaneous repair of UDT and hypospadias is rarely done. Herein, we present a novel technique to use processes vaginalis as a vascular cover for neourethra in a hypospadias patient with UDT. We have done urethroplasty and orchiopexy simultaneously. This is the first report concerning the use of processes vaginalis to reinforce the urethra.

**Aims:**

Simultaneous repair of hypospadias and undescended testis.

**Results:**

Both the patients withstood the procedure well. Postoperative period was uneventful. Patients passed urine in single stream without any fistula.

**Conclusions:**

In patients of undescended testis with hypospadias, simultaneous repair with the processes vaginalis flap is an ideal technique with good results. Processes vaginalis is good vascular cover for neourethra.

## INTRODUCTION

Repair of undescended testis with hypospadias is rarely done simultaneously and there are more chances of fistula formation as there is no adequate vascular cover for the neourethra. The objective of this technique was to find new surgical technique to achieve this aim.

## MATERIALS AND METHODS

Patient with undescended testis with hypospadias.

### Case 1

A 12-year-old boy presented with proximal penile hypospadias and bilateral palpable undescended testis. Patient had mild chordee and length of penis was adequate. Patient was evaluated for intersex and karyotype was 46 XY. Genitoscopy revealed evidence of large prostatic utricle. After release of chordee, tube pedicle urethroplasty (Duckett's) was done. The required 5-cm long tube was constructed on number 10 feeding tube. We routinely do proximal anastomosis first before making the tube.

**Figure 1:**
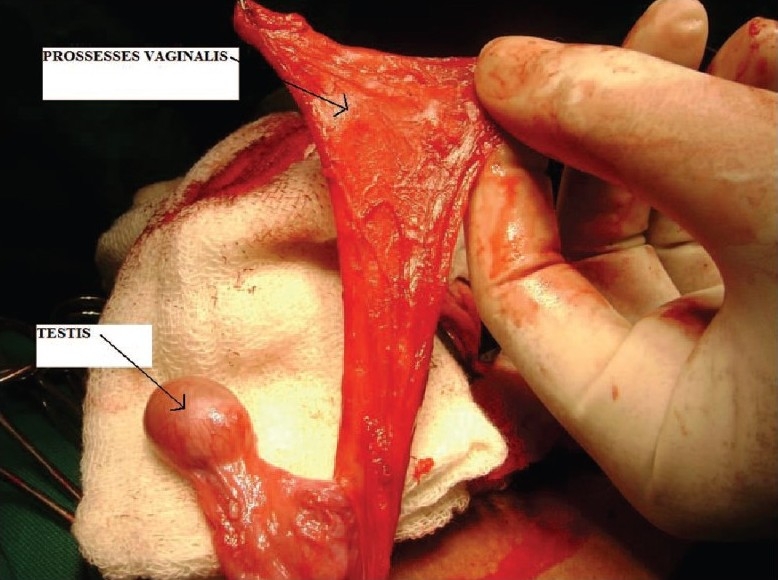
Prossess vaginalis dissected from testis

**Figure 2:**
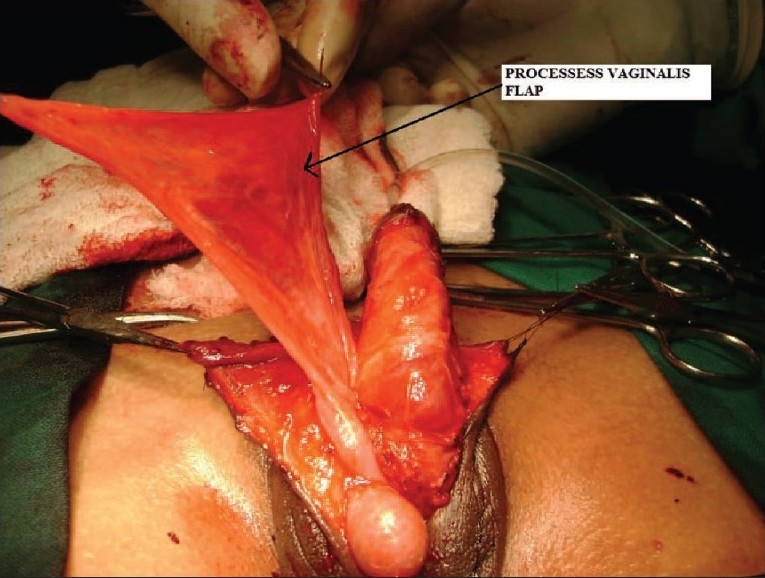
Testis brought in scrotum with prossess vaginalis flap

**Figure 3:**
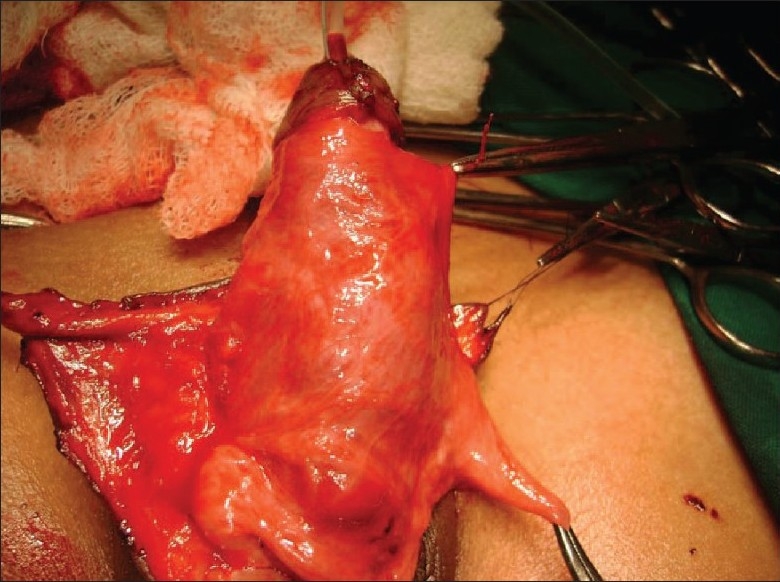
Prossess vaginalis wrap over neourethra

To prevent complications such as fistula, the urethral repair needs vascular cover. At this juncture, we planned to do orchidopexy along with the repair of hypospadias. Inguinal crease incision was taken on right side and herniotomy done close to the internal ring. After mobilizing the cord structure proximally, testis was brought down into scrotum along with its processes vaginalis [Figure 1]. Testis was fixed into extra dartos pouch and process vaginalis was cut open to form the flap [Figure 2]. This processes vaginalis was used to cover the neourethral repair as a blanket wrap [Figure 3]. The post operative course was uneventful and patient voided single good stream on tenth day of operation. Patient is on regular follow up with no complications.

### Case 2

A 3-year-old child of partial androgen insensitivity syndrome was operated by masculizing genitoplasty. Hypospadias repair was done with tube pedicle (ducketts).The repair was covered with processes vaginalis which was brought down along with left orchidopexy. Post operative period was uneventful and patient passed single stream urine on day 10 of operation. Patient is on regular follow-up without any complication.

## RESULTS

Both the patients stood the procedure well and after removal of catheter passed urine in single stream without any fistula. Patients are on follow-up for about six months with no complications.

## DISCUSSION

The incidence of the undescended testis along with hypospadias varies from 6% to 31%.[1] Urethrocutaneous fistula is the most common complication associated with the proximal hypospadias repair. There are various techniques to prevent the formation of fistula.

There are various methods for protective intermediate vascular cover to the neourethra to prevent fistula formation, Snow described the use of Tunica vaginalis wrap.[2] Retik was the first to use dorsal subcutaneous flap from the prepuce.[3] Motiwala described the use of Dartos flap from the scrotum.[4] Tunica vaginalis wrap is the most common method used with good results and long-term followup.[5]

In patients of hypospadias associated with the undescended testis, tunica vaginalis is not available to use as a protective layer for hypospadias repair. As scrotum is not well developed because of undescended testis, one cannot get easily the dartos fascia for cover of urethral repair.

Yamataka *et al*, had described method of using gubernaculum as vascular cover after dissecting it from the testis and leaving its anomalous distal attachment to the pubic tubercle intact, but the limitation of gubernaculum is that it is not long enough to cover whole urethra.[6]

We are presenting two cases where we could develop processes vaginalis flap and we could do orchidopexy and reinforce neourethra with processes vaginalis with good results. Our technique is useful to cover the anastomosis and suture line as well as allow for the simultaneous repair of UDT. Processes vaginalis is long and can be easily brought down with testis with good vascular supply. To achieve adequate mobilization of processes vaginalis, herniotomy should be done at internal ring level.

There are no reports in literature regarding the use of processes vaginalis as vascular cover and this simple technique will reduce the complication rate of hypospadias repair with UDT.
